# Peptide Selank Enhances the Effect of Diazepam in Reducing Anxiety in Unpredictable Chronic Mild Stress Conditions in Rats

**DOI:** 10.1155/2017/5091027

**Published:** 2017-02-09

**Authors:** Anastasiya Kasian, Timur Kolomin, Lyudmila Andreeva, Elena Bondarenko, Nikolay Myasoedov, Petr Slominsky, Maria Shadrina

**Affiliations:** ^1^Department of Molecular Basis of Human Genetics, Institute of Molecular Genetics, Russian Academy of Sciences, 2 Kurchatov Sq., Moscow 123182, Russia; ^2^Department of Chemistry of Physiologically Active Compounds, Institute of Molecular Genetics, Russian Academy of Sciences, 2 Kurchatov Sq., Moscow 123182, Russia

## Abstract

It was shown that the anxiolytic effect of Selank is comparable to that of classical benzodiazepine drugs and that the basis of their mechanism of action may be similar. These data suggest that the presence of Selank may change the action of classical benzodiazepine drugs. To test this hypothesis, we evaluated the anxiolytic activity of Selank and diazepam in rats both under conditions of unpredictable chronic mild stress and in its absence, after the individual and combined administration of these compounds using the elevated plus maze test. We found that, even in the absence of chronic stress, the administration of a course of test substances changed anxiety indicators toward their deterioration, but the changes after the administration of a course of Selank were less pronounced. In conditions of chronic stress, anxiety indicator values after the simultaneous use of diazepam and Selank did not differ from the respective values observed before chronic stress exposure. The data obtained indicate that the individual administration of Selank was the most effective in reducing elevated levels of anxiety, induced by the administration of a course of test substances, whereas the combination of diazepam with Selank was the most effective in reducing anxiety in unpredictable chronic mild stress conditions.

## 1. Introduction

A large number of stress factors of varying intensity influence people in modern society. Chronic stressful experiences lead to the development of neuropsychiatric disorders, especially anxiety disorders and depression [1].

Until recently, classical benzodiazepine drugs were widely used for the treatment of diseases such as neurosis, neurosis-like disorders (a group of neuropsychiatric disorders, which look like neurosis but are not caused by psychogenic effects, such as asthenic, phobic, monosymptomatic, motor, and somatic-vegetative syndromes), psychopathic conditions, and generalized anxiety disorders. Although these drugs have a strong protective effect under various stress loads, they have pronounced side effects. The basis of the mechanism of action of benzodiazepines (BZD), of which the reference representative drug is diazepam (DZ), is their ability to allosterically modulate GABA_A_ receptors, thereby amplifying the effects of the inhibitory neurotransmitter gamma-aminobutyric acid (GABA) in the central nervous system (CNS) [2].

Currently, anxiolytic drugs are used increasingly often in clinical practice, including those based on endogenous regulatory peptides that possess a wide spectrum of activity and minimal side effects and do not cause addiction and withdrawal syndrome [3]. One such drug is Selank, the structure of which includes a short fragment (Thr-Lys-Pro-Arg) of the human immunoglobulin G heavy chain, which was elongated at the C terminus via the addition of three natural L-amino acids (Pro-Gly-Pro) to enhance its metabolic stability and the length of action of the drug [4, 5]. Selank has a pronounced anxiolytic activity and sustained neuropsychotropic, antidepressant, and antistress effects and removes the reaction of aggression and fear [6–8].

Clinical studies have shown that the anxiolytic effect of Selank is comparable to the effect of low doses of the benzodiazepine tranquilizers; however, the action of Selank is not accompanied by the characteristic side effects of those drugs [9, 10]. Furthermore, Selank affects the specific binding of GABA to GABA_A_ receptors, which may be caused by a change in affinity of endogenous ligand receptors under the action of Selank [11]. This suggests that the presence of Selank may change the action of classical BZD.

To test this hypothesis, we evaluated the anxiolytic activity of Selank and DZ in rats subjected to unpredictable chronic mild stress (UCMS) after the single and combined administration of these compounds.

## 2. Methods and Materials

### 2.1. Chemicals

Dry preparations of Selank (N*α*-Thr-Lys-Pro-Arg-Pro-Gly-Pro-diacetate salt) and DZ (Sigma-Aldrich, USA) were dissolved to a concentration of 12 mg/ml and 20 mg/ml, respectively, in saline.

### 2.2. Animal Model

Male Wistar rats (Animal Breeding Facility-Branch of Shemyakin and Ovchinnikov Institute of Bioorganic Chemistry, Russia) with an average weight of 400 g were used in the experiment. The animals were housed under standard conditions with free access to water and food (when not under the UCMS stressor of food or water deprivation) and a 12 h light/dark cycle (daylight from 8:00 to 20:00). The rats were kept in groups of six per one cage. All animals were gently handled for 2 weeks before the start of the experiment (each rat was handled for 5 min each day).

The animal experiments were carried out in accordance with the National Institutes of Health *Guide for the Care and Use of Laboratory Animals* (NIH publication number 8023, revised 1978) and the statement of the ethics committee of the Institute of Molecular Genetics, Russian Academy of Sciences.

### 2.3. UCMS Protocol

The procedure used represents modified CMS protocol [12]. The animals (*n* = 48) were divided into 2 groups: a “rest” group (*n*_1_ = 24), which included animals that were not exposed to UCMS, and a “stress” group (*n*_2_ = 24), in which the animals were exposed to a combination of several types of mild stressors (food deprivation, water deprivation, 45° cage tilting, forced swimming at 12°C, change of the light/dark cycle, and damp sawdust) for 14 days. The sequence of stressors was intentionally designed to maximize unpredictability: the stressors were randomly scheduled over a 5-day period and repeated throughout the 2-week experiment. A design of the UCMS test is presented in Table 1. All animals were exposed to stresses for the same amount of time.

### 2.4. Administration of the Experimental Drugs

Animals from both groups (*n*_1_ and *n*_2_) were administered experimental substances once daily for 14 days. During this time, the animals from the “stress” group (group *n*_2_) were exposed to UCMS. Each of the test groups of rats was divided into 4 groups: control with administration of saline (*n*_1‐1_ = 6 and *n*_2‐1_ = 6) and 3 experimental groups with administration of Selank (*n*_1‐2_ = 6 and *n*_2‐2_ = 6), DZ (*n*_1−3_ = 6 and *n*_2‐3_ = 6), and DZ together with Selank (*n*_1−4_ = 6 and *n*_2−4_ = 6).

The animals in subgroups *n*_1‐1_ and *n*_2‐1_ were administered saline intranasally (5 *μ*l in each nostril) once daily; the animals in subgroups *n*_1-2_ and *n*_2-2_ were administered Selank dissolved in saline intranasally (at the rate of 300 *μ*g/kg; 5 *μ*l in each nostril); the animals in subgroups *n*_1–3_ and *n*_2-3_ were administered DZ dissolved in saline orally (1 mg/kg; 20 *μ*l of a solution once daily); the animals in subgroups *n*_1–4_ and *n*_2–4_ were administered Selank dissolved in saline intranasally (at the rate of 300 *μ*g/kg; 5 *μ*l in each nostril) and DZ dissolved in saline orally (1 mg/kg; 20 *μ*l of a solution). Selank dose of 300 *μ*g/kg was selected based on the data that this dose was the most effective therapy dose exerting anxiolytic action [10, 13]. The intranasal administration of Selank was shown to be optimal for the delivery of peptide molecules in the CNS [14, 15].

### 2.5. The Elevated Plus Maze (EPM) Test

The measurement of the behavior of animals was carried out using the “Elevated Plus Maze Test” equipment (OpenScience, Russia). The EPM consisted of a central area with the size of 14 × 14 cm, from which four arms (50 × 14 cm each) diverged crosswise at right angles. Two opposite arms were open and without boards, and two were closed and bound on each side by boards that were 30 cm high and painted in a dark color. The maze was elevated 80 cm above a floor.

Animals were tested twice: before beginning the administration of a course of the test substances and after this 14-day administration (24 hours after the UCMS and/or last administration of substances) (in accordance with the manufacturer's protocol). The animal was placed in the center of the EPM, facing the open arm. The time spent in the open (OA) and enclosed (EA) arms of the maze, the number of crossed squares, the number of rears, and the number of events of hanging from the OA were recorded. Each test lasted 3 min (in accordance with the manufacturer's protocol). The maze was cleaned with a 3% solution of diabak (INTERSAN-plus, Russia) after every trial to eliminate odor.

Video recording of animal behavior in the EPM test was performed on the video-tracking system GigE Vision (The Imaging Source, Germany). Videos were processed using a basic package of program RealTimer (OpenScience, Russia) for the planning and optimization of the experiment, behavior recording, and analysis of the results.

### 2.6. Statistical Analyses

Statistical data analyses were performed using Statistica 8.0 software, with the nonparametric Wilcoxon test for dependent samples (comparison of parameters before and after the administration of a course of substances to the same animal subgroups) and the Mann–Whitney test for independent samples (comparison of behavioral parameters between the groups). Statistical significance was set at *α* < 0.05.

## 3. Results

The evaluation of anxiety levels showed that, even in the absence of UCMS, the 2-week administration of the test substances to experimental rats changed anxiety indicators toward their deterioration (Table 2). Thus, after the administration of a course of saline to rats, the time spent in the open arms of the maze was reduced by 4.5 times, whereas in rats that were treated with DZ or a combination of Selank and DZ, this time was reduced by 5.7 and 5.4 times, respectively. It was noted that rats treated with saline were in the closed arms of the maze 1.6 times more often than before its course administration, and in the experiments with DZ and a combination of Selank and DZ, the residence time of the test animals in the closed arms increased by 1.3 and 1.6 times, respectively. In addition, in experimental rats that were treated with saline solution, the number of hanging events from the open arms of the maze was reduced by 4 times. Furthermore, statistically significant changes in the horizontal locomotor activity of animals after the administration of a combination of DZ and Selank were observed: the number of squares crossed was reduced by 2.6 times. Concomitantly, although the administration of a course of Selank led to a deterioration in anxiety indicators in rats, these changes were less pronounced compared with the similar changes observed in the other three subgroups of experimental animals (none of which were significant). Special attention should be paid to the fact that changes in the horizontal locomotor activity index (the number of squares crossed) were practically absent after the introduction of Selank.

The evaluation of anxiety indicators in rats that were exposed to UCMS showed that the deterioration of indicators was greater after the administration of a course of saline than it was in the subgroup of rats that were treated with saline solution in the absence of stress conditions. Thus, the residence time of test animals in the open arms of the maze decreased by 13.6 times; conversely, the duration of the stay in closed arms increased by 2.1 times (Table 3). In addition, the number of hanging events from the open arms of the maze decreased by 5.7 times in rats that were treated with saline solution after UCMS. Concomitantly, the application of any of the drugs for 2 weeks in the presence of UCMS did not lead to the significant deterioration of anxiety parameters that were observed after the introductory course of saline. Changes in the time spent in the open and closed arms of the maze in the presence of Selank under stress conditions did not differ from the changes observed during its administration to rats that were not exposed to UCMS. DZ administration during UCMS, compared with its use in the absence of stress, resulted in only a slight worsening of anxiety parameters: the length of stay in the open arms of the maze after stress exposure decreased by 2.1 times, and the residence time in the closed arms increased by 1.3 times. In the case of the simultaneous use of DZ and Selank, anxiety indicator values did not differ from the respective values observed before UCMS exposure. Furthermore, statistically significant differences in the time spent in the closed and open arms of the maze were found by comparing the indicators with the respective indicators in the animal behavior control group (treated with saline). The administration of a course of a combination of Selank and DZ resulted in a substantial improvement in these anxiety indicators: time spent in the open arms of the maze was 8.9 times higher than that observed during saline administration, and the residence time in the closed arms decreased by 2 times.

## 4. Discussion

Currently, several studies have reported the effect of UCMS on the levels of anxiety. Most of this research has shown that this type of stress leads to increased anxiety and locomotor activity [16], and only 2 studies have shown that UCMS has no effect on anxiety [17, 18]. It can be assumed that the ambiguous influence of this type of stress results from differences in the duration of its effect on experimental animals and combined elements of stress effects [16–19]. Experiments performed by Strekalova et al. in mice have shown that the exposure to 1 week of stress increases anxiety and motor activity at constant rates and that exposure to 4 weeks of stress decreases anxiety and increases motor activity [19, 20]. Based on the results obtained in the aforementioned studies, we chose a duration of 2 weeks for the UCMS test. We showed that, in the experimental rats that were exposed to stress factors for 2 weeks on the background of a course of saline, anxiety levels increased essentially, although statistically significant alterations in physical activity were not identified. Concomitantly, here we found that, even in the absence of UCMS, the administration of saline for 2 weeks resulted in an increase in animal anxiety. Thus, our results suggest that the course of administration of any substance in itself has a stress impact, which also probably contributes to the effects observed during the UCMS.

The study of the effect of the tested drugs on the characteristics of anxiety during UCMS and in its absence yielded ambiguous results. In the absence of UCMS, the administration of a course of test substances (as in the case of the administration of saline) caused an increase in anxiety, and effective anxiety-reducing effects were only observed when Selank was administered individually. In addition, in the cases of DZ and of its combination with Selank, the elevation in anxiety did not differ from that observed after the administration of a course of saline.

In the conditions of UCMS, as upon the administration of Selank and DZ, the anxiety level of experimental rats decreased compared with the level of anxiety observed in animals receiving saline solution, with greater reductions observed for the administration of a course of DZ. However, it should be noted that we did not record the total decline in the elevated levels of anxiety after the individual application of these medications. At the same time, the administration of courses of the combination of these drugs almost completely eliminated the effects caused by stress: anxiety levels did not differ from those observed before the UCMS, and their difference from the level of anxiety recorded in animals that received saline was statistically significant.

Our results confirm the similarity of the range of action of Selank and DZ and indirectly suggest that the molecular mechanisms of action of these drugs are similar. The anxiolytic effect of classical BZD, which include DZ, stems from their interaction with GABA_A_ receptors via benzodiazepine-binding sites [2]. In turn, Selank can influence the specific binding of GABA to GABA_A_ receptors, which may be caused by the modulation of the properties of the peptide, which apparently change the affinity of endogenous ligands during Selank exposure to the receptor [11]. The involvement of GABA_A_ receptors in the mechanism of action of Selank is also confirmed by the similarity in the changes of expression of the genes that are involved in neurotransmission in nerve cells in the frontal cortex of rats after the administration of the peptide and GABA [21]. Moreover, the results of the current study indicate that the combined administration of Selank and DZ amplifies their anxiolytic action; thus, it can be assumed that Selank not only modulates GABA_A_ receptors allosterically but also increases the affinity of DZ to these receptors.

Our findings agree well with the results of clinical studies, as it was shown that the administration of Selank to patients with a simple structure of anxiety and anxiety-asthenic disorders led to a therapeutic efficacy that was comparable to that of benzodiazepine anxiolytics (medazepam and phenazepam) without causing the undesirable side effects that are typical of these drugs, such as sedation, muscle relaxation, development of tolerance, and withdrawal syndrome [22, 23]. In addition, as shown by recent studies, the administration of Selank in combination with phenazepam for the treatment of anxiety disorders is probably able to enhance and hasten the onset of the therapeutic effect of phenazepam and reduce the severity of some of its side effects [24]. It should be noted that the tolerance was not observed after 14 days of Selank administration in the current study. Moreover, the combined use of Selank and DZ in the current study did not lead to an increase in motor activity, a decreased level of which was observed after a course of DZ. Despite the fact that the use of Selank in combination with DZ did not reduce its side effects, in the presence of Selank, the intensity of the anxiolytic action of DZ was significantly increased. We can assume that the use of a combination therapy of Selank and DZ will reduce the dose of the benzodiazepine drug, thereby reducing its adverse effects.

## 5. Conclusions

Thus, our findings indicate that the administration of a course of any substance in the absence of chronic stress by itself has a stress impact, leading to an increase in anxiety. The individual administration of Selank was the most effective in reducing elevated levels of anxiety, induced by the administration of a course of test substances, whereas the combination of DZ with Selank was the most effective in reducing anxiety in UCMS conditions. Moreover, our results support the hypothesis that allosteric modulation of the operation of the GABAergic system is one of the molecular mechanisms of action of Selank and suggest that the combination of Selank and BZD will reduce the dose of the drug, thus reducing its side effects.

## Figures and Tables

**Table 1:** Unpredictable chronic mild stress (UCMS). The text in bold denotes the start time of the respective types of mild stress exposure; the italicized text denotes the end of the exposure.

	Day 1	Day 2	Day 3	Day 4	Day 5	Day 6	Day 7	Day 8	Day 9	Day 10	Day 11	Day 12	Day 13	Day 14
Water deprivation	**16:00**	*10:00*				**16:00**	*10:00*				**16:00**	*10:00*		
Food deprivation		**14:00**	*14:00*				**14:00**	*14:00*				**14:00**	*14:00*	
Damp sawdust			**15:00**	*11:00*				**15:00**	*11:00*				**15:00**	*11:00*
Forced swimming				5 min										
Change of the light/dark cycle				**20:00**	*12:00*				**20:00**	*12:00*				
Cage tilting (45 degrees)					**17:00**	*10:00*				**17:00**	*10:00*			

**Table 2:** Indicators of the behavior of rats that were not exposed to chronic unpredictable stress at the EPM test before and after the administration of the test substances. 1, time spent in the open arms of the maze, s; 2, time spent in the closed arms of the maze, s; 3, number of hanging events from the open arms; 4, number of squares crossed; 5, number of rears.

Test substance		Saline	Selank	DZ	Selank + DZ
Behavioral measures		Ме (25%–75%)	Ме (25%–75%)	Ме (25%–75%)	Ме (25%–75%)
Measures of anxiety					
1	Before	48,9	40,2	51,4	42,3
		(42,5–109,7)	(16–68,2)	(20–87)	(18–78,4)
	After	**10,8** ^∗^	12,2	**9** ^∗^	**7,8** ^∗^
		(0–30,3)	(0–23,9)	(0–14,5)	(0–16,6)

2	Before	97,5	124,9	99,7	102
		(59,3–109,6)	(96,7–149,2)	(75,9–117,3)	(66,7–132,4)
	After	**152,3** ^∗^	147,7	**133,7** ^∗^	**162,8** ^∗^
		(135,3–167,8)	(127,8–172,2)	(113,3–177,2)	(147,4–170,9)

3	Before	8	7	10	8
		(7–11)	(3–9)	(6–11)	(4–10)
	After	**2** ^∗^	5	2	2,5
		(0–6)	(3–9)	(0–6)	(1–4)

Measures of locomotor activity					
4	Before	34	36	32	37,5
		(20–49)	(19–42)	(23–53)	(35–42)
	After	14	30	15,5	**14,5** ^∗^
		(11–57)	(17–42)	(9–21)	(12–21)

5	Before	8	9,5	7,5	6,5
		(7–9)	(8–13)	(5–10)	(6–10)
	After	5	9,5	6,5	6,5
		(3–10)	(9-10)	(5–8)	(5–8)

*Note*. Me: median; ^∗^*P* < 0.05, comparison of indicators before and after the administration of a course of the test substances (Wilcoxon's test).

**Table 3:** Indicators of the behavior of rats that were exposed to unpredictable chronic stress at the EPM test before and after exposure to stress and the administration of a course of the test substances. 1, time spent in the open arms of the maze, s; 2, time spent in the closed arms of the maze, s; 3, number of hanging events from the open arms; 4, number of squares crossed; 5, number of rears.

Test substance		Saline	Selank	DZ	Selank + DZ
Behavioral measures		Ме (25%–75%)	Ме (25%–75%)	Ме (25%–75%)	Ме (25%–75%)
Measures of anxiety					
1	Before	62,4	64,7	56,7	72,6
		(48,7–125,1)	(41,7–79,4)	(48,5–137,8)	(56,7–84,1)
	After	**4,6** ^∗^	**15,5** ^∗^	**26,9** ^∗^	**40,8** ^**#**^
		(0–17,8)	(7–26,6)	(8,5–47,7)	(25,3–57,5)

2	Before	78,2	104,7	99,1	72,9
		(42,3–113,3)	(57,3–120,6)	(34,2–108,1)	(65,5–99,2)
	After	**164,3** ^∗^	**139,9** ^∗^	**130,3** ^∗^	**83** ^**#**^
		(150,1–175,5)	(133,2–162,7)	(90,4–169)	(63,4–135,8)

3	Before	8,5	9	10,5	8
		(4–12)	(3–13)	(5–13)	(3–16)
	After	**1,5** ^∗^	4,5	6	9
		(0–4)	(3–6)	(2–9)	(8–10)

Measures of locomotor activity					
4	Before	23	39	25,5	40,5
		(23–47)	(33–59)	(14–36)	(23–49)
	After	27	22	16,5	25
		(21–34)	(15–30)	(12–26)	(22–38)

5	Before	8,5	6,5	7	7
		(6–12)	(5–10)	(5–12)	(6–12)
	After	5	5,5	7	5
		(4–8)	(3–8)	(5–10)	(3–7)

*Note*. Me: median; ^∗^*P* < 0.05, comparison of indicators before and after exposure to the stress and the administration of a course of the test substances (Wilcoxon's test); ^#^*P* < 0.05, comparison of indicators in the subgroup of animals with related indicators in the control group of animals (Mann–Whitney test).
